# Examining the Impact of Human Face Stimulus on Shape-Contrast Effects during a Brief Presentation

**DOI:** 10.3390/brainsci12070914

**Published:** 2022-07-13

**Authors:** Kun Qian

**Affiliations:** Institute for Asian and Oceanian Studies, Kyushu University, Fukuoka 819-0395, Japan; qk@kyudai.jp; Tel.: +81-92-802-6888

**Keywords:** visual illusion, visual aftereffect, face perception, shape-contrast effect

## Abstract

Shape-contrast effects have been introduced to the investigations into face perception with the aim of exploring face adaptation in the context of norm-based coding. Research has indicated that shape-contrast effects occur even for shapes as complex as the human face. However, whether the complexity of face stimuli alters the magnitude of shape-contrast effects needs to be examined. In this study, emoticons and realistic human faces were used with the original white circle as the test stimuli. The results revealed that the shape-contrast effect was dependent on the stimulus. However, there was no significant difference between the shape-contrast effect evoked by upright faces and that evoked by inverted ones. This suggests that the face stimuli influenced the strength of the shape-contrast effect: the mechanism of this effect involved multiple stages of the visual system related to luminance and complexity, rather than the holistic face perception.

## 1. Introduction

The visual aftereffects based on perceptual adaptation occur in several low-level visual attributes, such as contrast, size, color, orientation [1], texture [2], motion [3], and shape perception. The shape-contrast effect is a typical visual aftereffect of shape perception during brief presentations. This effect refers to the illusory distortion of geometric shapes caused by the priming stimulus. For example, a preceding presentation of a line flashing for 30 ms creates the appearance of a subsequent circle as elliptical, elongated, and perpendicular to the preceding line [4]. In the first report on the shape-contrast effect, this phenomenon was demonstrated with various presentation durations and geometric shapes [4]. They revealed that the exposure duration, luminance of stimuli, size of the priming line, spatial distance (i.e., eccentricity), and the temporal distance (i.e., stimulus-onset asynchrony) between the priming line and the subsequent circle all changed the strength of the shape-contrast effect. In addition to circular shapes, squares, triangles, and mouth-like curvatures were also examined as test stimuli. The authors claimed that shape-tuned neurons in the inferior temporal cortex (IT) mainly contributed to this effect. They concluded that the shape-contrast effect resulted from the process of early shape encoding. A subsequent study of convexity and concavity stimuli indicated that this type of perceptual adaptation also occurs during the perception of contour orientation, suggesting that the function of high-level neural activities encoding global configurations of orientation information also contributes to the shape-contrast effect [5]. The aftereffects of complex patterns with convexity or concavity were modulated by the weight of attention [6], and they were different from the simple patterns in their time duration for adaptation [7]. Three-dimensional objects (e.g., geometric cuboids or graphical heads) could reportedly generate a repulsive aftereffect after their perceptual rotation orientation [7]. A precise investigation using radial frequency patterns suggested that the shape-contrast effect was involved in the various stages of processing along the ventral pathway [8].

The IT plays a critical role in the ventral pathway during object perception [9]. IT neurons are selective for shapes, particularly during face perception [10], which is influenced by multiple visual attributes, such as facial color [11], background color [12], and perceptual aftereffects [13]. In addition to the IT, the MRI experiments have demonstrated that area V4 in the ventral pathway also contributes to visual aftereffects [14]. Face adaptation based on perceptual aftereffects has been previously demonstrated in several studies. Aftereffects occur in the context of face attractiveness [15], face identification [16], distortion of facial parts [17], personal familiarity [18], and basic characteristics of the face, such as sex, age, ethnicity, and expression [19]. The adaptability of norm-based coding of facial information has also been demonstrated [20,21]. Leopold et al. used realistic face stimuli as complex patterns to explore the neural mechanism of shape encoding. They found that even with complex shapes, such as faces, a perceptual adaptation and shape-contrast effect occurred because a briefly presented face significantly influenced and distorted the identification of a subsequent face [10]. It is clear that the shape-contrast effect occurs during complex face perception; however, to the best of my knowledge, whether the complexity of face stimuli alters the magnitude of the shape-contrast effect has never been investigated. In the present study, circular stimuli of three complexity levels were employed to test the magnitude of the shape-contrast effect. Complexity was defined as physical characteristics in this study, such as the number of patterns and variation of luminance in the stimuli. The white circle used in the original study [4] was considered the simplest stimulus, with uniform luminance and no other patterns included. Circularly trimmed realistic human faces (female and male) were used as the most complex stimuli. For the middle-level complexity, a monochrome emoticon was introduced into the experiment. An emoticon is a schematic face comprising basic geometric patterns, which is much simpler than a realistic face. Emoticons are widely used in text-based communication to deliver facial information linked to user identification and emotional expression [22,23]. However, the perception and recognition of emoticons involve different processes compared with realistic faces [24]. Even for the adaptation of facial expression, realistic and schematic faces produced different aftereffects, especially when facial parts were scrambled [25]. Emoticon faces have also been used to explore the aftereffects of happy and sad emotions by controlling the curvature of the mouth [26].

Based on the findings above, it is hypothesized that the shape-contrast effect involved in neural activity during multiple stages of visual cortex processing would be influenced by shape complexity. The present study predicted that the complex stimuli of the human face would influence the magnitude of visual aftereffects on shape perception. The realistic face stimuli were more detailed and complex, thereby producing stronger shape-contrast effects.

## 2. Experiment 1: Comparing the Shape-Contrast Effect Evoked by Emoticon Faces and Realistic Faces

### 2.1. Methods

#### 2.1.1. Participants

Seventeen healthy individuals with normal or corrected-to-normal visual acuity participated in the experiment (10 men and 7 women; mean age = 24.59 years; *SD* = 5.2). All the participants were naïve to the purpose of the experiment. The experiment in this study was approved by the Ethics Committee for Psychological Studies at the Institute of Decision Science for a Sustainable Society, Kyushu University, Japan (No. 2018/1–3). All experiments were performed in accordance with the relevant guidelines of the ethics committee. At the beginning of the experiment, participants provided written informed consent.

#### 2.1.2. Apparatus and Stimuli

The experiment was conducted using a computer (Apple, Mac Pro, MB871J/A, with Mac OS X 10.11.6, California, CA, USA) and a 21-inch CRT monitor (EIZO, FlexScan F931, 1152 × 870 pixels, 75 Hz, Hakusan, Japan). The experimental program was prepared and performed using PsychoPy (Version 3.1.2; [27]). The experiment was conducted in a dark room. The observers viewed the monitor binocularly with a chin and forehead rest at a viewing distance of 52 cm.

**Figure 1:**
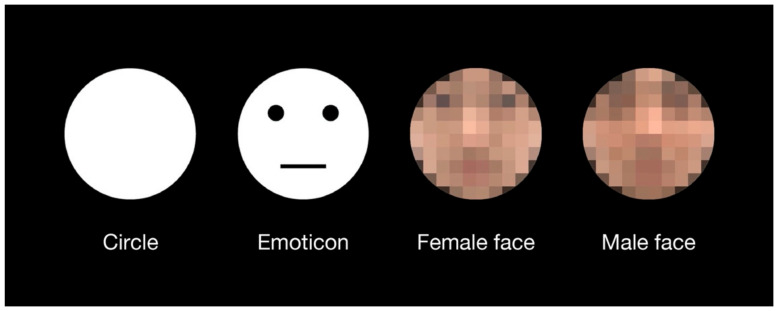
Test stimuli which were used in the experiment. An emoticon with a neutral expression was used because the expressions on the human faces were also neutral. Original images of a female face and a male face without the mosaic were used in the experiment. For this publication, they are presented as a mosaic version due to copyright protection of the facial expression database.

Four types of test stimuli were used in the experiment (Figure 1). The circle stimulus was a perfect white circle, which was the same as that used in previous investigations of the shape-contrast effect [4]. The emoticon stimulus was produced by adding simple geometric patterns—two black dots and one horizontal line—to the circular stimulus. Female and male face stimuli were selected from colored face samples with neutral expressions in a facial expression database [28]. All stimuli had the same area as the perfect circle at a 5.5° visual angle. There were no outlines of the test stimuli. The priming line presented in advance of the test stimulus was a white line with a length of 7.2° and a width of 2.2′ (one pixel). The fixation cross displayed at the beginning of each trial consisted of two orthogonal lines with a length of 35′ and a width of 4.3′. The response display consisted of a perfect circle with the same area as the test stimuli. The response circle was black with a white outline of 1-pixel thickness. The horizontal diameter of the response circle was adjustable according to participant response. The vertical diameter of the response circle was constant. All stimuli were displayed on a black background. The luminance values of black and white on the display were 1 cd/m^2^ and 94 cd/m^2^, respectively.

**Figure 2:**
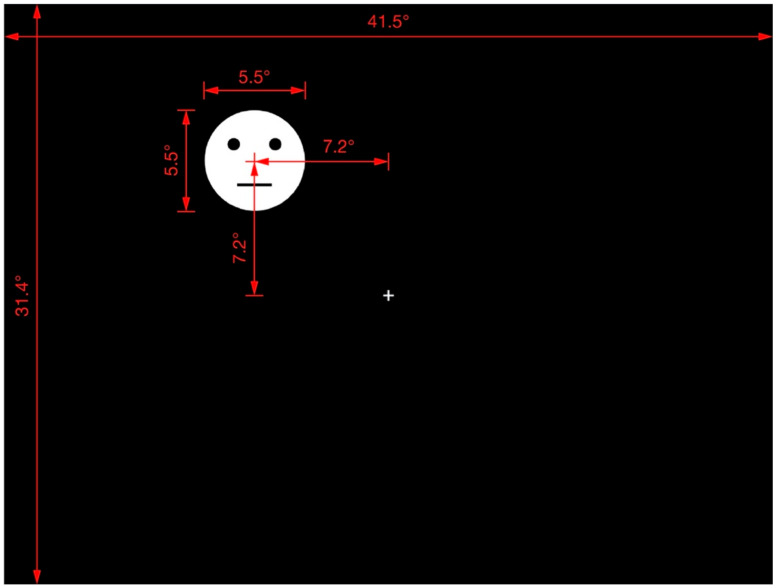
Display of test stimulus. Red lines, marks, and numbers were not presented in the actual experimental display. This is an example of the test stimulus appearing in quadrant II. The test stimulus and priming line could appear in any quadrant at the same distance from the center of the display.

The fixation cross and response circle were presented at the display center. The methodology of a previous study on the shape-contrast effect [4] was followed, and all test stimuli and their priming lines were presented randomly in one of the display quadrants. The distance between the center of the display and that of the test stimuli (or priming line) was 7.2 in both the horizontal and vertical orientations. Figure 2 provides further details of the arrangement and distances involved in the display of the test stimuli.

#### 2.1.3. Procedure

**Figure 3:**
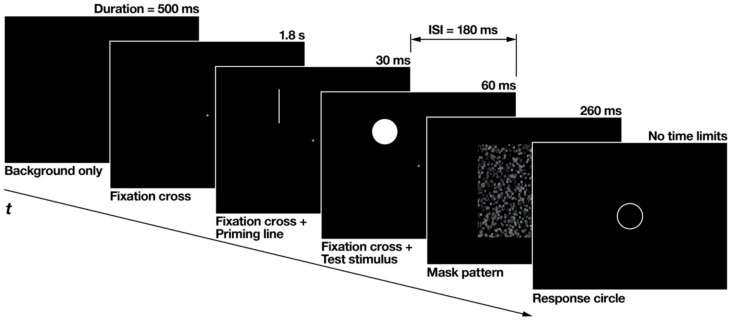
Flow of each experimental trial made from snapshots of the actual experiment displays. The thickness of the priming line and the response circle was enhanced for clearer definition. ISI is an abbreviation for inter-stimulus interval.

The flow of each experimental trial is illustrated in Figure 3. At the beginning of each trial, a black background was presented without any stimuli for 500 ms, followed by a fixation cross that was presented alone for 1.8 s. Subsequently, accompanied by the fixation cross, the priming line was displayed briefly for 30 ms. After an interstimulus interval of 180 ms, the test stimulus was flashed for 60 ms at the overlapping location of the priming line. After the test stimulus disappeared, a mask pattern of random black and gray squares was displayed for 260 ms. The fixation cross was continuously displayed until the mask pattern disappeared. The response circle appeared after the mask disappeared.

The participants were instructed to view the monitor by fixing their eyes on the fixation cross for as long as it was displayed. No fixation was required after the response circle appeared. The participants were asked to adjust the horizontal diameter of the response circle and to change the circle to resemble their perception of the test stimulus as much as possible using the right or left arrow keys. They were not required to respond rapidly. The next trial automatically started after each observer responded by pressing the space key.

Four types of test stimuli randomly appeared in one of the quadrants, yielding 16 experimental conditions; each condition was tested three times. A total of 48 experimental trials were conducted in a randomized order. Approximately 10 practice trials with test stimuli were randomly selected from the 16 conditions and conducted prior to the experiment.

#### 2.1.4. Data Analysis

The horizontal diameters of the response circles, which were adjusted by the participants, were automatically collected by PsychoPy. The perceived aspect ratio of the response circle was calculated using the adjusted horizontal and constant vertical diameters. The quadrants of the stimulus display were irrelevant to the shape-contrast effect, as suggested by previous research [4]. Thus, data from the perceived aspect ratios were averaged over the different quadrant displays. Three repetitions of the same experimental conditions were also averaged. The mean values and standard errors of the means were calculated for all 17 participants. A repeated-measures analysis of variance (ANOVA) with Tukey’s post hoc test was conducted on the mean perceived aspect ratios with the test stimuli factor.

### 2.2. Results and Discussion

**Figure 4:**
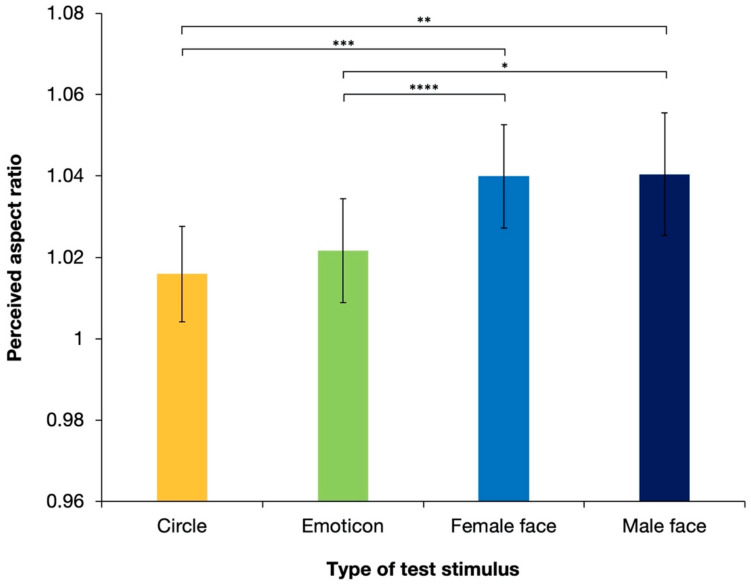
Results of the mean perceived aspect ratios of response circles averaged over 17 observers in Experiment 1. Error bars denote standard errors of the mean. The shape-contrast effect was strengthened significantly by a human face stimulus. Asterisks indicate significant differences resulting from multiple comparison test for the main effect of stimulus type; * *p* < 0.05, ** *p* < 0.01, *** *p* < 0.005, **** *p* < 0.001.

Figure 4 shows the aspect ratios of the perceived illusory ellipses, which were all physically perfect circles, with different types of test stimuli averaged over 17 observers. The perceived aspect ratios were >1 for all stimulus types, which suggested that the shape-contrast effect was reproduced in the experiment under all conditions. However, as shown in the figure, realistic faces (a “female face” and a “male face”) generated stronger effects than the other test stimuli. ANOVA revealed the significant effect of the stimulus type [*F*(3, 48) = 6.74; *p* < 0.001; partial η^2^ = 0.296]. Tukey’s post hoc test, based on the significant main effect (see details in Table 1), showed that the perceived aspect ratios were significantly different between the “circle” and “female face” stimuli, between the “circle” and “male face” stimuli, between the “emoticon” and “female face” stimuli, and between the “emoticon” and “male face” stimuli (*p*s < 0.05). There were no significant differences between the circle and emoticons (*p* = 0.405) or between female and male faces (*p* = 0.937).

These results revealed that shape-contrast effects occurred under all conditions. Illusory expansion was perceived in an orientation that was orthogonal to the priming line. The results also suggest that a realistic human face stimulus, regardless of sex, generated significantly stronger illusory expansion than the white circle or emoticon stimulus. Complex and detailed face stimuli enhanced the magnitude of the shape-contrast effect. However, one possible reason for the significant difference produced by the white circle or emoticon stimuli versus those with realistic faces is the difference in the color of the stimulus, and thus the luminance of the stimuli. In Experiment 1, the luminance of stimuli with realistic faces was lower than that of the white circle and emoticon stimuli. Previous research has suggested that lower luminance generates a stronger aftereffect. However, the luminance conditions employed in the present experiment were quite different from those in the experiments that previously suggested this [4]. To examine whether the strengthened aftereffects in Experiment 1 were due to the complicated cognitive processing of human faces or purely due to differences in luminance, Experiment 2 was conducted using upright and inverted faces. The perception of inverted faces is considered to require different processing from that of upright faces [29]. The effect of face perception on the shape-contrast effect will be denied if there are no significant differences between the shape-contrast effect produced by stimuli with upright faces and that produced by stimuli with inverted faces.

## 3. Experiment 2: Comparing the Shape-Contrast Effect Evoked by Upright and Inverted Faces

### 3.1. Methods

The methods were identical to those used in Experiment 1, except for the specifics described below.

Seventeen healthy individuals with normal or corrected-to-normal visual acuity participated in the experiment (7 men and 10 women; mean age = 24.71 years; *SD* = 8.3). The female and male face stimuli used in Experiment 1 and their inverted displays were employed as test stimuli in Experiment 2. In total, 48 trials were conducted in a randomized order in Experiment 2 (two genders of stimuli × two display patterns × two display patterns × four display positions × three repetitions).

### 3.2. Results and Discussion

**Figure 5:**
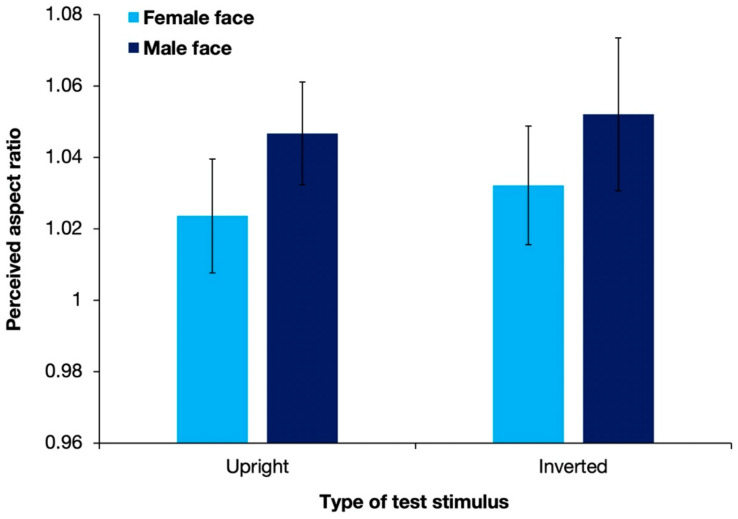
Results of the mean perceived aspect ratios of response circles averaged over 17 observers in Experiment 2. Error bars denote standard errors of the mean. Stimuli with a male face evoked a significantly stronger shape-contrast effect than stimuli with a female face.

Figure 5 shows the aspect ratios of the perceived illusory ellipses with different types of test stimuli, averaged over 17 observers. Replicating the results of Experiment 1, the perceived aspect ratios were >1 for all stimulus types. A two-way ANOVA revealed that the gender of the presented stimulus had a significant effect on the shape-contrast effect [*F*(1, 16) = 11.73; *p* = 0.003; partial η^2^ = 0.423]. However, neither the display pattern [*F*(1, 16) = 1.47; *p* = 0.244; partial η^2^ = 0.084] nor the interaction between the two factors [*F*(1, 16) = 0.05; *p* = 0.823; partial η^2^ = 0.003] had a significant effect.

In contrast with the results of Experiment 1, stimuli with a male face were found to produce a significantly stronger shape-contrast effect than stimuli with a female face. One reason behind the discrepancy might be that without stimuli of white circles, the luminance difference between male and female faces could be detected more precisely. Based on informal observations, the male face was slightly lower in luminance than the female face. The distinction of the slight difference in luminance between stimuli with male faces and stimuli with female faces was inhibited in Experiment 1 when compared with stimuli of a much higher luminance. However, there was no significant difference in the shape-contrast effect evoked by stimuli with upright faces and stimuli with the inverted faces, suggesting that the complicated cognitive processing of holistic face perception was not the key reason for a stronger shape-contrast effect.

## 4. General Discussion

In this study, Experiment 1 revealed that the exposure to realistic face stimuli generated stronger shape aftereffects than schematic face or non-face stimuli. The complexity of the test stimuli modulated the magnitude of the shape-contrast effect during a brief presentation. The information processing of complex shapes, such as realistic faces, was relevant for higher-level functions of the visual cortex. Furthermore, the results of Experiment 2 suggest that the complexity of information processing cannot be accounted for with the special processing required for holistic face perception, since there was no significant difference in the shape-contrast effect evoked by upright faces and that evoked by inverted faces.

Some previous studies have provided evidence that shape aftereffects are related to a higher-level perceptive or cognitive progress. Shape aftereffects occur on the path of apparent motion, which implies that the neural representation of apparent motion generated by higher cortical area feedback interfered with the lower-level coding of the stimulus shape [30]. Visual adaptation is also involved in the sex specification of the human body [31], realistic landscape scene categorizations [32], emotional responses to complex pictures [33], and in the detection and recognition of ambiguous [34] and novel objects [35]. Furthermore, some cross-category adaptations have been demonstrated. For example, face adapters induce aftereffects on body stimuli [36]. Geometric frames of image skew generate visual adaptation to natural scenes [37]. All of these visual adaptations and incidental aftereffects imply that shape aftereffects occur in complex contexts of shape perception as a function of the lower to higher cortical areas of the visual pathway. According to neurophysiological experiments, other than IT, other cortical areas, such as V4, are crucial for shaping adaptation processes in the visual cortex [14]. Therefore, it is proposed that higher-complexity stimuli engage in higher cortical functions in shape perception, consequently resulting in stronger shape aftereffects.

However, when discussing the results of the present study, the “shape” aspect and “face” aspect should be differentiated from each other. Dennett et al. proposed that face aftereffects arising from multiple levels of the visual system should be subdivided into face-level and shape-generic components. They found face distortion aftereffects useful for investigating face space, but it is difficult to minimize or account for the shape-generic contribution [38]. In addition, although it was clear that the complexity of face stimuli strengthened the shape aftereffect in the present study, it was difficult to determine which component was more effective and critical. When mentioning shape-generic contributions, especially for stimuli using realistic objects, including faces and shape constancy, operations should also be considered, because shape aftereffects can be influenced by the perceived shape, which is determined by constancy operations [39]. Shape constancy also provides evidence that shape aftereffects involve higher-level neural substrates.

The limitation of this study is that even though the results revealed that complex stimuli resulted in stronger aftereffects, the functions of complexity and luminance were not demonstrated independently. Thus, it is still uncertain whether low-level processing (i.e., luminance) or a high-level one (i.e., complexity) was the key reason for the shape-contrast effect. As advised by an anonymous reviewer, an algebraical model should be introduced in future studies to investigate how multiple functions from lower to higher visual processing account for the generation of this effect. Finally, the role of complexity revealed in the present study will be a useful factor for exploring other mechanisms of visual phenomena or illusions, in addition to shape-contrast effects. In contrast with the preceding visual cue (i.e., the priming line used in the present experiment), the succeeding contexts also modulated the perception of a briefly presented test stimulus in the opposite manner [40]. This so-called assimilation effect occurs even when the position of the test stimulus and the following stimulus differ [41]. The other related visual illusion was the egg illusion, where perfectly circular white patches located within gray bars were perceived as elliptical, similar to the appearance of a shape-contrast effect [42]. Recent research on the egg illusion has revealed that reducing geometric components from the grid configuration significantly weakens the illusory effect [43,44]. This result could be explained by the correlation between complexity and visual aftereffects found in the present study, because reducing geometric components also decreases the complexity of the global configuration.

## Figures and Tables

**Table 1:** Results of post hoc comparisons. Significant differences are revealed between human face stimuli and geometric pattern stimuli.

Comparison		Mean Difference	*SE*	*df*	*t*	*p*	*p*_tukey_
Circle	- Emoticon	−0.864	1.03	48	−0.841	0.405	0.835
	- Female face	−3.599	1.03	48	−3.504	0.001	0.005
	- Male face	−3.680	1.03	48	−3.583	<0.001	0.004
Emoticon	- Female face	−2.736	1.03	48	−2.663	0.011	0.050
	- Male face	−2.817	1.03	48	−2.742	0.009	0.041
Female face	- Male face	−0.081	1.03	48	−0.080	0.937	1.000

## Data Availability

The data presented in this study are available on request from the corresponding author. The data are not publicly available due to restrictions on privacy.
